# Engaging stimulatory immune checkpoint interactions in the tumour immune microenvironment of primary liver cancers – how to push the gas after having released the brake

**DOI:** 10.3389/fimmu.2024.1357333

**Published:** 2024-02-19

**Authors:** Yannick S. Rakké, Sonja I. Buschow, Jan N. M. IJzermans, Dave Sprengers

**Affiliations:** ^1^ Department of Surgery, Erasmus MC-Transplant Institute, University Medical Center, Rotterdam, Netherlands; ^2^ Department of Gastroenterology and Hepatology, Erasmus MC-Cancer Institute-University Medical Center, Rotterdam, Netherlands

**Keywords:** immune checkpoint stimulation, immunotherapy, hepatocellular carcinoma, cholangiocarcinoma, immunoglobulin superfamily, tumour necrosis factor receptor superfamily, receptor super clustering, bispecific antibody

## Abstract

Hepatocellular carcinoma (HCC) and cholangiocarcinoma (CCA) are the first and second most common primary liver cancer (PLC). For decades, systemic therapies consisting of tyrosine kinase inhibitors (TKIs) or chemotherapy have formed the cornerstone of treating advanced-stage HCC and CCA, respectively. More recently, immunotherapy using immune checkpoint inhibition (ICI) has shown anti-tumour reactivity in some patients. The combination regimen of anti-PD-L1 and anti-VEGF antibodies has been approved as new first-line treatment of advanced-stage HCC. Furthermore, gemcibatine plus cisplatin (GEMCIS) with an anti-PD-L1 antibody is awaiting global approval for the treatment of advanced-stage CCA. As effective anti-tumour reactivity using ICI is achieved in a minor subset of both HCC and CCA patients only, alternative immune strategies to sensitise the tumour microenvironment of PLC are waited for. Here we discuss immune checkpoint stimulation (ICS) as additional tool to enhance anti-tumour reactivity. Up-to-date information on the clinical application of ICS in onco-immunology is provided. This review provides a rationale of the application of next-generation ICS either alone or in combination regimen to potentially enhance anti-tumour reactivity in PLC patients.

## 1 Introduction

Primary liver cancer (PLC), including hepatocellular carcinoma (HCC) and cholangiocarcinoma (CCA), is the third leading cause of cancer-related death ranking sixth in incidence worldwide (1). Its incidence is expected to increase in Western society (2, 3). Early- or intermediate-stage HCC can be treated successfully using ablative therapy, surgical resection, or liver transplantation while early-stage CCA can be treated using surgical resection only. Moreover, the majority of PLC patients get diagnosed at advanced-stage disease leaving them to no other option than systemic therapies including immune checkpoint inhibitors (ICI) and multi-tyrosine kinase inhibitors (TKIs; e.g., sorafenib, regorafenib) for HCC and chemotherapy for CCA (e.g., cisplatin/gemcitabine, FOLFIRINOX) (4, 5). So far, these remaining treatment options for advanced-stage PLC have only shown modest survival benefits and more effective treatment approaches are urgently needed. Next to ICI, engaging immune (co-)stimulatory molecules (i.e., immune checkpoint stimulation (ICS) in the tumour microenvironment alone or combined with other immune enhancing therapies represents a promising novel opportunity. In this review, we first summarise the current knowledge on ICI and its pitfalls in the hepatic tumour immune microenvironment (TIME). Then we provide an overview of insights gained from (pre)clinical studies regarding the interactions between co-stimulatory molecules and their ligands expressed on different T-cell subsets, antigen presenting cells and other cell types in the context of the hepatic TIME. Lastly, we highlight the opportunities to enhance and support currently applied ICI and more targeted immune therapies using ICS in PLC.

## 2 Immune checkpoint inhibition fails to induce anti-tumour immunity in the majority of primary liver cancer patients

ICI is often applied in the form of antibodies that interfere with binding of co-inhibitory receptors (i.e., cytotoxic T-lymphocyte-associated protein 4 (CTLA-4), and programmed cell death-1 (PD-1)) and their cognate ligands (CD80/86 and programmed cell death ligand-1 (PD-L1), resp.). ICIs have been shown to effectively enhance pre-existing anti-tumour immune-responses among multiple immune-active cancer types such as melanoma, non-small cell lung cancer, and renal cell carcinoma (6–8).Accordingly, anti-PD1 antagonistic antibodies (pembrolizumab and nivolumab, resp.) prolonged survival in advanced-stage HCC patients that did not respond well to TKIs only. However, clinical efficacy of anti-PD1-mediated ICI was modest and failed to sustain its benefit when compared to TKIs directly upon randomisation among TKI-naive HCC patients (9, 10). Combination regimen of various ICIs or chemotherapy with ICI appeared to be more successful. Anti-PD-L1 antagonistic antibodies (atezolizumab) with anti-vascular endothelial growth factor (anti-VEGF; bevacizumab) improved overall and progression-free survival (OS and PFS) outcomes compared to the TKI sorafenib (11, 12). Similarly, sintilimumab (anti-PD-1) plus IBI305 (bevacizumab biosimilar) improved survival rates compared to sorafenib in Chinese patients with unresectable, hepatitis B virus (HBV)- associated HCC (13). Moreover, durvalumab (anti-PD-L1) in combination with the anti-CTLA-4 agonistic antibody tremelimumab improved OS compared to sorafenib (14). As atezolizumab-bevacizumab (atezo-bev) and durvalumab-tremelimumab (durva-trem) were proven to successfully induce clinical anti-tumour efficacy, both regimens have been approved by the U.S. Food and Drug Administration (FDA) and European Medicines Agency (EMA) to treat unresectable HCC patients in the first line of care. Also, in refractory or recurrent CCA, a combination regimen of ICI with chemotherapy was superior to chemotherapy alone. In the TOPAZ-1 trial, durvalumab with gemcibatine plus cisplatin (GEMCIS) improved both OS and PFS compared to chemotherapy with placebo (15). Based on these data durva-GEMCIS has been granted FDA-approval as first-line standard-of-care in advanced-stage CCA.

Still, even though these immunotherapy combination regimens have proven to successfully induce anti-tumour immunity objective responses could only be confirmed in 20-27% of HCC patients and about 27% of CCA patients (11, 13–15). This data underlines the complexity of the hepatic tumour immune microenvironment (TIME) potentially explaining inter-patient variation regarding clinical efficacy. Establishment of a suppressive TIME may enable tumour cells to evade and restrain efficient anti-tumour immunity (16). Compared to adjacent tissue compartments, the hepatic TIME has been shown to be enriched specifically for immune suppressive cells (e.g., regulatory T cells (Tregs), myeloid-derived suppressor cells (MDSCs), tumour-associated macrophages (TAMs)), rather than immune effector cells (17–19). Furthermore, in HCC and CCA, tumours can be either *inflamed* or *non-inflamed*, but the latter is the dominant phenotype in both settings indicating tumour-immunity may also often be poorly developed (20, 21) Indeed, the anti-tumour adaptive immunity in PLC has been shown to be hampered by impaired T cell priming and local dysfunction or exhaustion of tumour-infiltrating lymphocytes (TILs) (22, 23).

**Figure 1:**
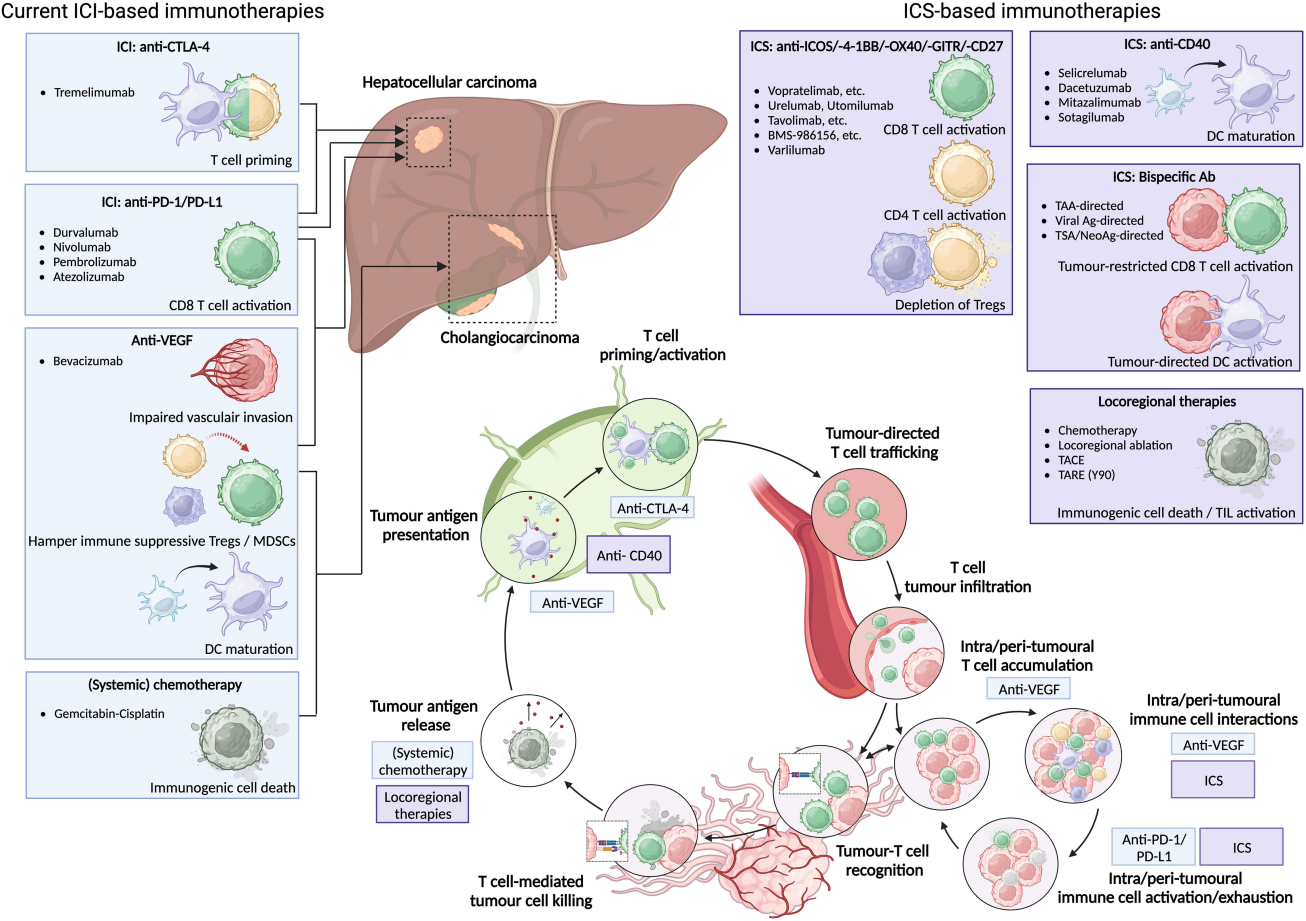
Current ICI-based immunotherapies for HCC and (i)CCA can be supported by ICS-based immunotherapies to enhance anti-tumour immunity. Current ICI-based immunotherapies for HCC consist of anti-PD-1/PD-L1 monotherapy, anti-CTLA-4 plus anti-PD-1/PD-L1, anti-PD-L1 plus anti-VEGF; or anti-VEGF plus Gem-Cis for CCA. Novel ICS-based immunotherapies can either provide additional CD4 and CD8 T cell activation, induce depletion of immunosuppressive Tregs, or induce DC maturation. Moreover, ICS-based bispecific antibodies might induce tumour-directed DC activation as well as tumour-restricted CD8 T cell activation and subsequent tumour cell killing via tumour-associated antigen (TAA), viral antigen (viral Ag), or tumour-specific/neoantigen (TSA/neoAg). All can be enhanced by additional release of tumour antigen via locoregional therapies.

The notion that combination regimens have proven to be clinically more effective compared to monotherapy in PLC suggests that successful reinvigoration of anti-tumour immunity in PLC might need a multi-factorial approach. Whereas ICI primarily intents to enhance existing cytotoxic CD8 T cell (CTL)-immune effector function, PLC-directed immunotherapies may require inhibition of immune suppressive cells as well. With respect to the latter, bevacizumab has been described to inhibit MDSCs, TAMs, and Tregs and these effects could partly explain its enhanced anti-tumour activity in the atezo-bev regimen (24) (**Figure 1**). Likewise, combination regimes of ICI with ICS using either agonistic antibodies or ligands targeting costimulatory molecules, could enhance T cell-mediated anti-tumour immunity. These ICS may act through depletion of immune suppressive cells as well as by enhancing the priming and activation of immune effector cells. Thereby, ICS might provide an interesting alternative or supportive immune therapeutic approach in PLC (25). Recent early phase I clinical trials have reported on the clinical safety and efficacy of ICS used alone or in combination regimes in various advanced solid tumours (26–30). To now develop more effective combination regimes based on ICI and ICS for the treatment of PLC, a comprehensive overview of the different stimulatory checkpoint mechanisms that might support intra-tumoural T-cell responses and their interplay with immune inhibitory checkpoint mechanisms in PLC is required and this we aim to supply with this review.

## 3 Co-stimulatory immune checkpoints are widely expressed among liver-resident innate and adaptive immune subsets

Stimulatory immune checkpoint interactions are crucial for effective T cell activation. In 1987, CD28 was the first co-stimulatory receptor that was demonstrated to enhance T cell receptor (TCR) signaling, thereby laying the foundation for the *three-signal model of T cell activation* that requires both TCR and co-stimulatory signaling as well as cytokines for full T cell activation and differentiation (31). Following recognition of the cognate peptide-MHC complex by the TCR, co-signaling receptors co-localise with TCR molecules at the immunological synapse. In contrast to co-inhibitory immune checkpoint interactions that provide feedback inhibitory signals to activated CTL in the effector phase, co-stimulatory interactions rather are important during antigen presentation, priming, and subsequent T cell activation or differentiation (25, 32) (**Table 1**). Co-stimulatory receptors are divided into two distinct groups: immunoglobulin superfamily (IgSF) members and tumour necrosis factor receptor superfamily (TNFRSF) members, and these subclasses are in turn divided according to protein structure and function (25).

**Table 1:** Immune co-stimulatory receptors as divided by IgSF and TNFRSF members are expressed among different immune cell subsets playing various roles in functional T cell engagement.

Receptor family	Receptor subfamily	Receptor molecule	Receptor expression		Ligand	Ligand expression	Functional T cell engagement					
			Immune cell subsets	Pattern			Priming	Expansion	T(h) differentiation	Effector	Survival	Memory
IgSF	CD28	CD28 (Tp44)	CD4^+^ (naive/activated) T cell CD4^+^ FoxP3^+^ T cell CD8^+^ (naive/activated) T cell	Consitutive	***B7.1 (CD80)***, ***B7.2 (CD86)***, B7H2 (ICOSL/B7RP1/CD275)	***DC*** ***B cell*** Macrophage ***Monocyte***	+	+		+	+	+
IgSF	CD28	ICOS (CD278/CVID1)	***CD4^+^ (activated) T cell*** ***CD4^+^ FoxP3^+^ T cell*** CD8^+^ (activated) T cell	Inducible	B7H2 (ICOSL)	***DC*** B cell Macrophage		+	+	+	+	+
IgSF	CD226	CD226 (DNAM1)	***CD4^+^*** (activated) ***T cell*** ***CD4^+^ FoxP3^+^ T cell*** ***CD8^+^ PD1^-/int^ T cell*** NK cell Monocyte	Constitutive	CD112, CD155	DC Monocyte Fibroblast Endothelial cell ***HCC cancer cell***		+	+	+		
IgSF	TIM	TIM-1	CD4^+^ T cell CD8^+^ T cell NK cell ***B cell*** Macrophage DC Mast cell	Inducible	TIM-4, phophatidylserine	NKT cell B cell Mast cell		+	+	+		
IgSF	SLAM	CD2	CD4^+^ T cell CD8^+^ T cell NK cell Thymocyte DC	Inducible	CD58 (LFA3)	CD4^+^ T cell CD8^+^ T cell B cell Monocyte Granulocyte Thymic epithelial cell	+	+		+		+
IgSF	SLAM	SLAM-6 (NTB-A)	CD4^+^ T cell CD8^+^ TFC-1^+^ T cell NK cell B cell	Inducible	SLAM-6	CD4^+^ T cell CD8^+^ TFC-1^+^ T cell NK cell B cell			+	+		
TNFRSF	Type-V	4-1BB (CD137/TNFRSF9)	CD4^+^ (activated) Treg CD4^+^ (activated) Th CD4^+^ FoxP3^-^ ***CD8^+^ CD39^+^ CD103^+^ PD1^hi^***	Inducible	4-1BBL (CD137L)	DC B cell Macrophage CD4^+^/CD8^+^ T cell NK cell Mast cell Smooth muscle cell Haematopoietic progenitor cell		+		+	+	+
TNFRSF	Type-V	OX40 (CD134)	***CD4^+^ (activated) Treg*** ***CD4^+^ (activated) Th cell*** CD4^+^ FoxP3^-^ T cell ***CD8^+^ T cell*** NK cell NKT cell Macrophage	Inducible	OX40L (CD134L)	DC B cell Macrophage CD4^+^/CD8^+^ T cell NK cell Mast cell Endothelial cell Smooth muscle cell	+	+	+	+	+	+
TNFRSF	Type-V	GITR (CD357/TNFRSF18)	***CD4^+^*** (activated) ***Treg*** **CD4^+^** (activated) ***Th*** CD4^+^ FoxP3^-^ ***CD8^+^*** PD1^int/hi^ ***T cell*** B cell NK cell	Inducible	GITRL (CD357L/TNFSF18)	DC B cell Macrophage Endothelial cell		+		+		
TNFRSF	Type-V	CD27 (TNFRSF7)	CD4^+^ T cell CD4^+^ FoxP3^+^ T cell ***CD8^+^ T cell*** ***B cell*** NKT cell ***NK cell***	Constitutive (Other); Inducible (B cells)	CD70	DC B cell CD4+/CD8+ T cell NK cell Mast cell Endothelial cell Smooth muscle cell	+	+		+	+	+
TNFRSF	Type-V	HVEM (CD270)	CD4^+^ T cell CD4^+^ FoxP3^+^ T cell CD8^+^ T cell DC NK cell Monocyte Neutrophil	Inducible	LIGHT, BTLA, CD160, LTα3	B cell CD4^+^/CD8^+^ T cell	+	+		+	+	+
TNFRSF	Type-L	CD40 (TNFRSF5)	CD8^+^ T cell ***B cell*** ***DC*** Macrophage Cancer cell	Inducible	CD40L	CD4^+^ (activated) Th CD8^+^ T cell Basophil Mast cell	+					

All ICS receptors that have been described in PLC among the various immune cell subsets are depicted in bold and curse. DC, dendritic cell; GITR, glucocorticoid-induced TNFR-related; HVEM, herpes virus entry mediator; ICOS, inducible T cell co-stimulator; IgSF, immunoglobulin superfamily; PD1, programmed death-1; NK, natural killer; SLAM, signaling lymphocyte activation molecule; TIM, topical immune modulation; TNFRSF, tumour necrosis factor receptor superfamily.

### 3.1 IgSF structure, expression, and ligands

The IgSF comprises various cell surface and soluble proteins. Its members share structural features with immunoglobulins, including an Ig-domain. IgSF members include cell surface antigen receptors, cell adhesion molecules, cytokine receptors, and co-inhibitory or -stimulatory signaling receptors. The human co-stimulatory IgSF members consists of 12 receptors and 16 ligands that are represented in the CD28, CD226, TIM, and CD2/SLAM receptor subfamilies (25). We here highlight the most relevant subfamily members.

#### 3.1.1 CD28 receptor subfamily: CD28

**Figure 2:**
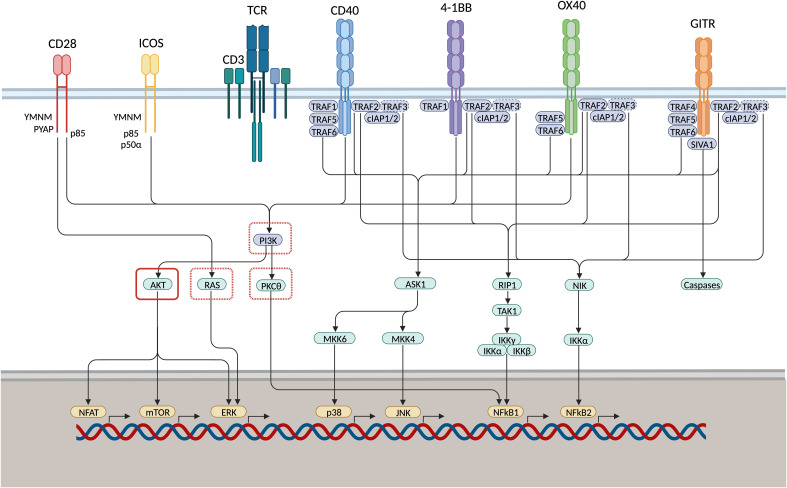
IgSF and TNFRSF members induce T cell proliferation, survival, and effector function via shared co-stimulatory signaling pathways. CD28 and inducible T-cell co-stimulator (ICOS) associate with phosphoinositide 3-kinase (PI3K) through their YMNM/PYAP- or YMFM-motif, respectively. TNF receptor monomers multimerise into trimeric ligand-receptor complexes that engage TNF receptor-associated factor (TRAF) adaptor proteins. Upon ligation, GITR associates with TRAF2/4/5/6, 4-1BB associates with TRAF1/2, OX40 associates with TRAF2/5/6, CD40 associates with TRAF1/2/5/6. p38 and JNK are activated subsequent to TRAF2 and -5-mediated regulation of the MAPK pathways. NfkB is induced via the canonical and non-canonical signaling cascade. NFkB1 is induced through activation of kinase RIP1, TAK1 and IKK complexes mediated via TRAF2 or TRAF5 association. Moreover, TRAF2 engages with TRAF3 via cIAP1/2, thereby inducing TRAF3 degradation. As TRAF3 mediates NIK degradation under natural conditions, NFkB2 activation is induced downstream of phosphorylation of the inhibitory kappa B kinase-alpha (IKK) as a result of NF-kB-inducing kinase (NIK) stabilisation. GITR associates with pro-apoptosis factor SIVA1, activating downstream caspases. This might function as a negative feedback loop to pro-survival signaling cascades vai Bcl-xL. PI3K, RAS, and PKC𝜃 are inhibited through PD-1 signaling (red dashed square) directly or via recruitment of protein tyrosine phosphatase SHP1. AKT can be inhibited through PD-1 and CTLA-4 signaling (red solid square) via recruitment of SHP1 or protein phosphatase 2A (PP2A), respectively. Therefore, co-stimulatory signaling pathways might be enhanced through concomitant application of both ICS and ICB.

CD28 (alias: Tp44) is a co-stimulatory molecule that was first described in 1987 (31, 33). Generally, CD28 is expressed constitutively on CD4^+^ and CD8^+^ T cells, including those in the TIME. Expression has been demonstrated among bone marrow stromal cells and other immune subsets such as plasma cells, neutrophils, and eosinophils (34). CD28-directed ligands belong to the B7 family of which CD80 (alias: B7-1) and CD86 (alias: B7-2) demonstrate the highest binding affinity (Kd: 4uM and 15-40uM, resp.) (25, 35, 36). CD80 and 86 are upregulated on APCs upon activation and maturation following immune stress responses. CD28 signaling induces activation of NFAT, mTOR, ERK and NFkB lowering the threshold for TCR signaling and subsequent T cell activation and proliferation, survival and effector function (**Figure 2**). However, in the TIME, CD80/86-mediated CD28 signaling may be hampered due to competitive binding of PD-L-1 and CTLA-4 to their respect receptors (37). Both PD-1 and CTLA-4 signaling impair CD28 signaling as intracellular domains of activated or PD-L-1 bound PD-1 bind to the CD28 cytoplasmic tail with high affinity. Moreover, CD28-mediated T cell co-stimulation has been shown to be crucial for PD-1 therapy in cancer patients as loss of CD28 via T cell exhaustion was shown to correlate to clinical irresponsiveness towards PD-1 ICI (37).

In HCC, CD28 has been described to be expressed on TILs albeit at lower levels compared to PBMC-derived T cell (38). This might be explained by the fact that TIL fractions are enriched for central and effector memory T cells (Tcm and TEMRA, resp.) rather than naïve CD8 T cells (Tn). Alternatively, diminished CD28 may be a reflection of T cell exhaustion (37). Among CD3^+^ TILs, Hsu and colleagues have demonstrated high levels of PD1/CD28-coexpression (39). Furthermore, CD28 is upregulated on CD8^+^ PD1^hi^ HCC TILs compared to the PD1^-^ and PD1^int^ compartments thereby potentially delineating tumour-reactive TILs (40). In the TIME multiple cell types can deliver CD28 stimulatory signals as CD80 and CD86 are expressed on intra-tumoural B cell, BDCA1^+^ myeloid DC (mDC), and CD14^+^ monocytes in both HCC and CCA (18). HCC tumour cells themselves, however, have demonstrated relatively low B7 family member expression (41, 42).

As CD28 has been described to be expressed constitutively on naive T cells, agonistic targeting of CD28 is hard to restrict to the TIME or to circulating tumour-specific T cells. This was illustrated by a first-in-human clinical trial of a CD28 agonistic antibody where all healthy volunteers developed life-threatening immune-related adverse events (irAE) as a direct result of a cytokine release syndrome (43). Direct CD28 stimulation of TILs does, however, did show potential *in vitro*, reinvigorating anti-tumour CD8 TIL function and metabolic activity (44). Alternatively, CD28 stimulation might rather be established indirectly through antibody-mediated blockade of anti-PD-L-1 and CTLA-4 allowing increased binding of CD80/86 to CD28 and/or by lifting of inhibitory signaling via PD-1 and CTLA-4. Especially in HCC the former might contribute to the effect of ICI therapies targeting PD-L-1 and CTLA-4, since HCC tumour cells of early-relapsed disease demonstrated enhanced interaction of PD-L-1 and CTLA-4 with CD80/86 compared to primary HCC tumour cells (45). In these cases anti-PD-1/CTLA-4 blockade might enhance CD28 signaling and thereby could support T cell activation by local antigen presentation, thus improving tumour immune surveillance.

#### 3.1.2 CD28 receptor subfamily: ICOS

Engagement of CD28 will trigger most T cells. To specifically target only already activated cells using ICS, one might focus on activation-induced co-stimulatory receptors such as inducible T-cell co-stimulator (ICOS; aliases: CD278, CVID1) (46) (**Figure 2**). Only a small fraction of resting memory T cells shows expression of ICOS at low levels (25). ICOS expression has been demonstrated to be expressed at intermediate levels in immune-active tumour-infiltrating Th1 cells (47). Furthermore, both in colorectal cancer (CRC) and non-small cell lung cancer (NSCLC), Duhen and colleagues demonstrated tumour-reactive CD4^+^ Th or follicular T helper (Tfh) TILs co-express PD1 and ICOS (48). Important to consider when targeting ICOS is that in the TIME ICOS may also be highly expressed on FoxP3^+^ Tregs, identifying highly immune suppressive activated Tregs among ICOS^hi^ fractions (47, 49). Hence, stimulating ICOS may also augment immune suppression via Tregs. The ligand for ICOS is B7H2 (aliases: ICOSLG, B7RP1, CD275) (50). ICOS and CD28 share the B7H2 ligand, although ICOS binds to B7H2 with significantly higher affinity. B7H2 is expressed constitutively on mature APCs (e.g., B cells, macrophages, and DCs) (50). Moreover, B7H2 is largely expressed on somatic cells such as tumour cells including HCC, under local control of TNFα (51, 52). Similar to CD28, agonistic ICOS engagement triggers the activation of multiple pathways that support antigen specific T cell activation (**Figure 2**).

Upregulation of ICOSL on HCC tumour-derived plasmacytoid DCs (pDCs) was hypothesised to activate type 1 regulatory T (Tr1) cells only (53). However, in HCC TILs, ICOS is upregulated both on Tregs as well as on CD4^+^ TILs that demonstrated features of recent activation and displayed increased proliferative capacity as well (54–57). Therefore, ICOS signaling in HCC might both enhance and hamper local tumour control by liver-resident immune cells. In contrast, CCA-derived intra-tumoural Tregs demonstrated high expression of ICOS but not effector Th or CTL (19).

Consistent with these expression patterns, in preclinical studies, ICOS signaling either promoted pro-tumour responses (via Tregs) or anti-tumour responses (via Th1, Tfh, or CTL) (47). Hence, agonistic ICOS mAbs alone will most likely not achieve any anti-tumour activity due to predominant action on Treg subsets. Interestingly, in patients treated with anti-CTLA-4 mAb, ICOS appears to be upregulated on effector T cells (58). Moreover, ICOS knock-out mice do not respond well to anti-CLTA-4, suggesting a significant role for ICOS-signaling on effector T cell-mediated anti-tumour activity particularly when Tregs are blocked (59). Concordantly, in a murine tumour model, concomitant stimulation of ICOS and blockade of CTLA-4 has proven to elicit potent synergistic anti-tumour responses, pleading for clinical exploration of combination regimen of ICOS stimulation with ICI, especially anti-CTLA-4 antibodies (60, 61). Future research may focus on antibody engineering to enhance the potential of ICOS^hi^ Treg depletion via antibody-dependent cytotoxicity and also the timing of Treg depletion with respect to ICOS stimulation. On another note, ICOS stimulation has recently been combined with PD-1 blockade. Yap and colleagues reported on a phase 1/2 trial on vopratelimab (humanised IgG1 agonistic ICOS antibody; alias: JTX-2011) combined or not with nivolumab in refractory advanced-stage solid tumours, including 2 CCA patients in the monotherapy arm (62). Though vopratelimab alone or in combination with nivolumab was tolerated well, objective response rates (ORRs) were only 1.4% and 2.3% respectively.

#### 3.1.3 CD226, TIM, and CD2/SLAM IgSF subfamily members as potential targets for ICS

CD226 (alias: DNAM1, TLiSA1) is a constitutively expressed co-stimulatory receptor that was discovered first in 1985 (63). Being expressed mostly by effector T cells, Tregs, and NK cells, CD226 regulates immune activity via interplay with its ligands CD155 and CD112.

At the tumour site CD226 expression tends to be diminished due to PD-1 and TIGIT signaling, local regulation through TGF-ß, and proteasomal cleavage (64). Given the significant immunostimulatory role of CD226 via VAV1, agonistic CD226-targeting mAbs could potentially serve as promising anti-tumour regimen (65, 66). However, CD226 plays a significant role in blood platelet adhesion and activation as well, potentially complicating its clinical application. T cell Ig and ITIM domain (TIGIT) competes with CD226 for binding CD155 (Kd 114-119nM vs. 1-3nM) and CD112 (Kd 0.31-8.97uM vs. not measureable) (67). In HCC-derived CTL, Th, and Treg TILs the TIGIT/CD226 ratio appeared to be upregulated (68). Therefore, enhancing CD226 signaling specifically on TILs might be achieved indirectly using selective blockade of TIGIT. Indeed, low PD1 expressing CD8^+^ T cells reacted to anti-PD-1 and anti-TIGIT combination regimen in a CD226-dependent manner *in vitro* whereas these CTLs did not respond to anti-PD-1 alone. Concordantly, in the setting of NSCLC, Banta, et al. demonstrated the importance of CD226 expression for optimal anti-tumour CD8^+^ T cell responses in the context of therapeutic ICI via PD-1 or TIGIT (69).

TIM-1 is a co-stimulatory receptor that is part of the IgSF TIM subfamily. TIM-1 ligands are TIM-4 and phophatidylserine. TIM-1 expression is induced upon activation of T cells, NK cells, B cells, macrophages, DCs, and mast cells (70). In a mouse transplant model, agonistic TIM-1 mAb have shown to stimulate effector T cell function and deprogram Tregs (71). In HCC, TIM-1 expressing B cells have been demonstrated to delineate immune suppressive subsets. TIM-1 targeting might therefore have a stimulatory effect by deprogramming Bregs as well (72). Stimulating effector T cells and hampering regulatory subsets, TIM-1-mediated ICS might be a potential candidate for enhancing anti-tumour activity (73). However, recently TIM-1 expression by cervical cancer cells was associated with cancer proliferation and migration indicating harmful effects may also arise. The expression of TIM-1 on liver tumour cells is unknown, hence more studies are required before agonistic targeting of TIM-1 can be applied in the clinic (74).

SLAMF6 (alias: NTB-A) is expressed on B, NK, and T cells as well. Remarkably, SLAMF6 expression is strongly correlated to the expression of T cell factor 1 (TCF-1) (75). Both are described to delineate progenitor exhausted CD8^+^ T cells from terminally-exhausted CTLs, making SLAMF6-mediated ICS of great interest in rescuing tumour-specific CTLs (76). SLAMF6 has neither been characterised in HCC nor in CCA. However, in HCC progenitor exhausted CD8^+^ T cells have been demonstrated *ex vivo* by PD1^int^, TCF-1^+^, TOX^lo^ expression, providing a potential reservoir for SLAMF6-mediated ICS to reinvigorate anti-tumour immunity (68).

### 3.2 TNFRSF structure, expression, and ligands

The human TNF-super-family (TNFSF) consists of 29 receptors and 19 ligands. Upon ligation, TNF receptor monomers multimerise into trimeric ligand-receptor complexes that engage TNF receptor-associated factor (TRAF) adaptor proteins (**Figure 2**). TNF-/TNF-super-family members are divided into four separate categories of which the type-V (divergent) and type-L (conventional) play a prominent role in stimulatory T cell co-signalling (25). To date, 4-1BB, OX40, GITR, CD27, and CD40 are considered appropriate candidates for therapeutic immunomodulation.

#### 3.2.1 Type V TNFSF receptor subfamily: 4-1BB

The activation-induced co-stimulatory molecule 4-1BB (aliases: TNFRSF9, CD137, ILA) was first described in 1989 (77). 4-1BB is transiently expressed upon TCR engagement on TILs, including activated CD8^+^ T cells (78–80), memory and regulatory CD4^+^ T cells (81), follicular CD4^+^ T (Tfh) cells (82), but also on NK cells (83). Additionally, 4-1BB expression by TILs is enhanced partly under hypoxic conditions through hypoxia-inducible factor 1-α (79). 4-1BB binds uniquely to its 4-1BB ligand (4-1BBL; aliases: TNFSF9, CD137L). 4-1BBL is expressed on antigen presenting cells such as dendritic cells (DCs), B lymphocytes, and macrophages (84, 85). Furthermore, upon inflammation, non-immunological human cells (e.g., smooth muscle cells, endothelial cells, hematopoietic stem cells) demonstrate expression of 4-1BBLas well, suggesting a role for these cells in effector T cell enhancement (86, 87). Besides a membrane-bound form (m4-1BB), 4-1BB also exists in a soluble form (s4-1BB) that results from alternative splicing (88). Although initially s4-1BB was observed in patients with autoimmune disease, increased levels have been demonstrated in haematological malignancies as well (89). Moreover, s4-1BB is hypothesised to function as a cancer immune escape mechanism by competing with m4-1BB for binding to 4-1BBL, thereby hampering intratumoural 4-1BB T cell costimulation (90).

In HCC and intrahepatic CCA, 4-1BB has been described to be exclusively expressed by CD4^+^ and CD8^+^ TILs (91, 92). Interestingly, Kim and colleagues have shown that 4-1BB delineates a distinct activation status among exhausted PD1^hi^ CD8^+^ HCC-derived TILs (92). Compared to 4-1BB^-^ TILs, 4-1BB^+^ PD1^hi^ CD8^+^ T cells expressed higher levels of TCF-1, CD28, and T-bet/Eomes, indicating a greater potential for TIL reinvigoration. Accordingly, co-stimulation of CD8^+^ TILs using a humanised IgG4 4-1BB mAb further reinvigorated anti-PD-1-mediated CD8 function *in vitro*. As 4-1BB^+^ PD1^hi^ CD8^+^ TILs are hypothesised to be tumour-reactive T cells, 4-1BB costimulation using agonistic antibodies may be promising anti-tumour strategy in HCC patients (92). Care should be taken, however, as 4-1BB has also been demonstrated to be expressed highly on Tregs in colorectal cancer-derived liver metastasis (CRLM), as well as on TIL-derived Tregs in HCC (93, 94). Nevertheless, pre-clinical mouse studies have revealed a potential of dual anti-tumour activities of 4-1BB mAb by which both Treg depletion and CD8 T cell promotion can be achieved by smartly exploiting antibody isotypes and FcγR-availability (95).

Care should be taken though as in 2008, high doses of urelumab (alias: BMS-663513), a fully humanised IgG4 mAb targeting 4-1BB, led to two hepatotoxicity-related deaths. Lower dosage regimens, however, appeared to be safe in later studies in hematological cancer patients and were accompanied by CR/PR of 0%/6%, 6%/6%, and 17%/0% in DLBCL, FL, and other B cell lymphomas, respectively (26) (96) In addition, Utomilumab (alias: PF-05082566), a fully humanised IgG2 mAb, has been studied as a single agent, engaging 4-1BB to mediate T cell ICS. It was well-tolerated safety profile among 55 patients (MCL, CRC, GC, PDAC, (N)SCLC, CCA, BC, Lymphoma, Sarcoma, etc.) (97). When combined with pembrolizumab, no dose-limiting toxicities were observed and 6 out of 23 patients demonstrated CR or PR (2 CR: RCC, SCLC; 4 PR: TC, RCC, NSCLC, and HNSCC) (98). Interestingly, clinical activity correlated with increased levels of peripheral activated memory/effector CD8^+^ T cells. Also, other combination regimen of utomilumab with mogamulizumab (anti-CCR4) or avelumab (anti-PD-L1) appeared to be safe, but anti-tumour activity remained relatively small (99, 100). Based on these experiences a very diverse landscape of second-generation 4-1BB agonists has recently been developed, with many entering clinical Phase 1 and 2 trials (101).

#### 3.2.2 Type V TNFSF receptor subfamily: OX40

OX40 (aliases: TNFRSF4, CD134) is a co-stimulatory molecule that was discovered in 1987 by Paterson and colleagues (102). OX40 expression can be induced following TCR cross-linking on activated CD4^+^ and CD8^+^ T cells, and Tregs. Moreover, it is induced upon the activation of NK cells, NKT cells, and neutrophils (103–105). OX40 is overexpressed on T cells upon sustained TCR stimulation in the presence of CD28-mediated costimulation as well as IL2 (106). *In vitro*, OX40 gets upregulated on CD4^+^ and CD8^+^ TILs when exposed to autologous tumour cells (107). Accordingly, OX40 appears to be expressed at higher levels in TILs or tumour-draining lymph nodes when compared to PBMC-derived immune cells in melanoma, ductal mamma carcinoma, and head and neck cancer (108–112). OX40 ligand (OX40L; alias: TNFSF4, gp34) functions as the unique ligand for the OX40 receptor (113). OX40L is expressed transiently on antigen presenting cells such as DCs, B lymphocytes, and macrophages upon ligation of certain pattern recognition or cytokine receptors (e.g., TLR2, TLR4, TLR9, CD40, TSLPR, IL18R) (114–117). Moreover, T cells have been demonstrated to upregulate OX40L themselves as a result of TCR crosslinking upon T-T cell interactions, giving rise to sustained CD4^+^ T cell longevity (118). Innate-derived immune cells such as NK cells and type 2/3 innate lymphoid cells (ILC) express OX40L when triggered by NKG2D or alarmin molecules, respectively (119, 120). As reported for 4-1BBL, non-immunological cells (endothelial cells, smooth muscle cells) can express OX40L under inflammatory conditions as well (121, 122). Though, the soluble variant of OX40L (sOX40L) is increased in some types of cancer, it cannot oligomerise and hence sOX40L does not properly stimulate OX40 (123).

In HCC, OX40 was found enriched in the TIME (124–127). Expression was enhanced specifically among Treg and CD4^+^ activated helper T cell (aTh) TIL subsets (124, 126). Ligation of OX40 markedly increased CD4^+^ T cell expansion *in vitro* (126). CD8^+^ TIL fractions, in contrast, showed only modest expression levels of OX40 (126). However, OX40 was largely co-expressed with exhaustion marker PD1 on CD8^+^ T cells from HCV-related HCC patients, suggesting prior antigen specific activation and thus hinting to pre-existing *in situ* anti-tumour reactivity (124). Moreover, OX40 correlated to higher expression of other immune-activation markers such as: CD68, TIM-3, and LAG3 (125). Though, OX40 expression is overall associated with anti-tumour immunity, in HCC it has also been correlated to more aggressive disease (i.e., displaying increased alpha feto-protein (AFP) and vascular invasion) and to impaired survival (125). In contrast, in CCA, increased expression of OX40 on PBMC-derived Th and CD8^+^ T cell was rather correlated to improved recurrence-free survival, hinting to a potential anti-tumour effect (128). Despite the association of OX40 to more aggressive disease in HCC, Treg, Th, and CTL in HCC-derived TILs can be skewed to the pro-inflammatory state upon multimerisation of OX40 using a hexameric OX40 ligand or bead-bound or Fc-engineered OX40 antibody *in vitro*. From these experiments it was suggested that FcγR(IIB)-mediated antibody multimerisation, to allow for OX40 trimerisation, is critical to effectively induce OX40-mediated anti-tumour immunity (126). Clinical trials on OX40 stimulation so far, however, have all applied traditional agonistic mAb with insufficient FcγR(IIB) affinities.

In a first attempt, a therapeutic agonistic mouse mAb to OX40 demonstrated tumour regression in 12 out of 30 late-stage cancer patients (129). Interestingly, in this study regression and SD were observed among patients with CCA. However, induction of human anti-mouse antibodies made re-administration of the agent impossible. More recently, Tavolimab (alias: MEDI0562), a humanised agonistic OX40 IgG1 antibody, was deemed safe in advanced-stage solid tumours but PR was observed in only 2 out of 50 patients, not comprising the single HCC patient included (130). Nonetheless, a marked increase in proliferation of peripheral CD4^+^ and CD8^+^ memory T cells was observed. Moreover, intra-tumoural FoxP3^+^ T cells decreased. Also, another fully humanised agonistic OX40 IgG1 antibody (BMS-986178) was shown to be safe in metastatic solid tumours (131). Clinically meaningful anti-tumour efficacy was not observed among any of the HCC patients included in this trial. Similar safety profiles on OX40 monotherapy have been observed in other recent clinical trials studying ICAGN01949 (fully humanised agonistic OX40 IgG1 mAb), GSK3174998 (fully humanised agonistic IgG1 mAb), or ivuxolimab (alias: PF-04518600; fully humanised agonistic IgG2 mAb) in advanced solid tumours and ivuxolimab in AML, respectively (132–135). Interestingly, one HCC patient, a non-responder to prior sorafenib treatment, demonstrated long-term tumour regression on ivuxolimab (132). A 30mg flat dose of ivuxolimab is currently evaluated in an expansion trial for efficacy, safety, and pharmacodynamics in HCC patients specifically. In patients treated with ICAGN01949 also one patient with metastatic CCA receiving 700mg demonstrated PR as best response with a largest decrease in tumour size of 41.9% from baseline (133). OX40 ligation may also hold promise in the neoadjuvant setting as was suggested by a recent study applying tavolimab pre-operably to HNSCC patients. In most patients enhanced immune activation in peripheral CD4^+^ and CD8^+^ T cells was observed and 4 out of 17 patients displayed expansion of putative tumour reactive CD103^+^CD39^+^CD8^+^ TILs. Importantly, in contrast to immune non-responsive patients, none of these patients developed recurrent disease (29).

Because of the modest efficacy of OX-40-mediated ICS, combination regimens have been clinically tested as well. BMS-986178 alone did not show any objective response in advanced-stage solid tumours. In combination with nivolumab and/or ipilimumab (anti-CTLA-4), ORR ranged from 0 to 13%, but did not surpass expected ORRs for anti-PD-1/CTLA-4 monotherapies (28). A second trial studying the combination of anti-OX40 antibodies with ICI (tavolimab vs. tavolimab with durvalumab (anti-PD-L1) or tremelimumab (anti-CTLA-4)) again demonstrated no improved clinical activity compared to monotherapy ICI (136). Combination therapies of multiple ICS strategies involving OX40-engagement (i.e., ivuxolimab with utolimumab and GSK3174998 with GSK1795091) do show anti-tumour immune reactivity. These trials, however, lacked a monotherapy arm (137, 138). Taken together, a clear clinical benefit using agonistic mAb to OX40 has not yet been demonstrated but there is remaining clinical potential for future FcγR(IIB)-binding antibodies.

#### 3.2.3 Type V TNFSF receptor subfamily: GITR

Glucocorticoid-induced TNFR-related protein (GITR) (aliases: TNFRSF18, CD357, AITR) is a co-stimulatory molecule that was firstly described in 1997 (139). Tregs demonstrate constitutive high expression levels of GITR, whereas naive and memory TILs have lower expression levels (140, 141). Upon T cell activation via CD28 signalling, GITR expression can be enhanced rapidly in both Treg and effector TILs (140). GITR is expressed transiently at low to intermediate levels in B cells, and innate lymphocyte subsets such as macrophages, NK cells, and NKT cells as well (142–144). GITR binds uniquely to GITR ligand (GITRL; aliases: TNFSF18, CD357L) (145). GITRL is expressed on activated APCs such as macrophages, DCs, and B cells (143, 146, 147). GITRL has been demonstrated to be transiently upregulated upon TLR4 activation (148). Moreover, GITRL is expressed by endothelial cells (145, 149). It is therefore hypothesised to play a role in mediating leukocyte adhesion and migration.

In HCC, TIL-derived (CD4^+^CD25^+^ or CD4+FoxP3+) Tregs have enhanced GITR expression compared to Tregs in adjacent tissues or PBMC (150) (55). *In vitro*, co-culture of HCC-derived CD4^+^CD25^-^ Th cells and CD4^+^CD25^+^ Tregs showed that GITR ligation prevented hypo-responsiveness of effector CD4^+^ T cells, suggesting GITR engagement either hampered Tregs or stimulated Th cells directly (55). Even though GITR is most abundant on activated CD4+ Tregs, it is also detected on CD4^+^ Th and CD8^+^ effector TILs (151–153). Interestingly, these TILs demonstrated co-expression of GITR with other activation induced checkpoint inhibitors and stimulators such as CTLA-4, PD1, and 4-1BB offering opportunity for combination therapies (151, 152). When GITR ligation was combined with CTLA-4-mediated ICI *in vitro* on HCC-derived TILs, immunosuppression by tumour-derived Tregs was abrogated completely (151). in addition, HCC-derived CD8^+^ TILs were functionally enhanced when GITR ligation was combined with PD-1 blockade, further paving the way for combination therapies (92, 152). Similar results were obtained for intra-hepatic cholangiocarcinoma (iCCA) where CD4^+^ Tregs also demonstrated higher GITR expression compared to CD4^+^ Th, and CD8^+^ effector cells (19). Additionally, *in vitro* GITRL enhanced proliferation of both pre-stimulated CD4^+^ and CD8^+^ TILs compared to anti-PD-1- or -CTLA-4-mediated ICI. Interestingly, in CCA GITRL is downregulated on mDCs and monocytes in TILs when compared to adjacent liver tissues (19). Thereby, potentially hampering stimulation of anti-tumour TIL activity. Similar to CD4^+^ T cells, CD8^+^ TILs largely co-expressed GITR with other checkpoint molecules underscoring the potential of combination-therapies in both CCA and HCC.

Clinical activity of GITR-mediated ICS using TRX-518, a humanised agonistic GITR glycosylated IgG1 mAb, was evaluated in 43 patients with refractory solid tumours including 1 HCC, 1 fibrolamellar, and 1 CCA patient (30). TRX-518 was safe and depleted peripheral Tregs. Nevertheless, patients developed neither PR nor CR. CD8^+^ T cell exhaustion was hypothesised to cause clinical inactivity, pleading for combinatorial approaches using PD-1-mediated ICI. Similarly, MK-1248, a humanised agonistic GITR IgG4 antibody had no clinical effect as monotherapy for patients with solid cancers, including 1 HCC patient (154). However, when combined with pembrolizumab 1 and 2 out of 17 patients developed CR and PR, respectively (HNSCC, melanoma and cancer e.c.i.). In contrast, in an interim-analysis of a phase 2 trial on advanced solid tumours, no additional clinical activity of GITR engagement using BMS-986156 (humanised agonistic GITR IgG1 mAb) to nivolumab was demonstrated (2 and 19 out of 252 CR and PR, resp.) (27) Still, 5 out of the 12 HCC patients in the combination arm experienced radiological disease control of which four had SD and one had PR. In ICI-naïve but not ICI experienced melanoma patients, MK-4166 (humanised agonistic GITR IgG1 mAb), significantly increased the ORR (5 and 3 out of 13 CR and PR, resp.). Moreover, combination of MK-4166 with pembrolizumab did not result in enhanced clinical activity as was observed in the single HCC patient that got doublet therapy (155). In accordance, GWN323, a humanised agonistic GITR IgG1 mAb, appeared to be safe, but showed minimal clinical activity as monotherapy and modest clinical benefit in combination with spartalizumab (anti-PD-1 mAb) in both advanced solid tumours as well as lymphomas (156). In conclusion, GITR appears to be an attractive target for ICS *in vitro*. In light of the importance of multimerisation of TNFRSF like OX40 the focus should maybe be shifted towards novel strategies to deliver adequate GITR-mediated ICS such as multimerisation approaches, to potentially reproduce advantageous results *in vivo* as well.

#### 3.2.4 Type V TNFSF receptor subfamily: CD27

In contrast to other TNFRSF members, CD27 (alias: TNFRSF7) is expressed constitutively on naive and effector T cells. This suggests that CD27 may act earlier upon activation in priming of T cells. Though CD27 gets downregulated on effector phenotypes, it is still expressed at moderate levels by central- and effector-memory T cells. NK cells also express CD27, albeit at lower levels in activated subsets. Moreover, CD27 is expressed by germinal centre and memory B cells (157, 158). It ligates specifically to CD70 (alias: TNFSF7, CD27L) that is expressed upon activation of DCs, B cells, and T cells.

In HCC tumours CD27 was found expressed on tumour-infiltrating B and T cell but the majority of CD27^+^ TILs were CD3^+^ T cells (159, 160). Compared to healthy controls, circulating CD27^+^CD19^+^ B cells in HCC appeared to be decreased especially with disease progression (161). Accordingly, expression of CD27 on T and B cells has been shown to be associated positively with patient survival (159, 162). CD27 has been demonstrated to be co-expressed with CD38 in HCC tumours, potentially delineating NK cells (159). Concordantly, a fraction of HCC-derived liver-resident NK cells expressed CD27. Additionally, NK cell CD27 was downregulated upon increased tumour burden which corresponded with impaired cytotoxic capacity *in vitro*, suggesting CD27 expression may associate with prognosis (163).

Besides T cell priming and effector differentiation, continuous ligation of CD27 is considered to play a significant role in the induction of T cell exhaustion as well as Treg survival. Nevertheless, CD27 agonistic antibodies exhibit anti-tumour functionality in both pre-clinical *in vitro* and *in vivo* models by enhancing CD27-mediated ICS and depletion of Tregs via ADCC (164, 165). Agonistic targeting of CD27 to stimulate anti-tumour T cell reactivity has been studied on a smaller scale when compared to other ICS-mediated T cell engagement. Treatment of 25 (Melanoma, CRC, OC, PC, RCC, NSCLC) and 31 patients (melanoma, RCC), with varlilumab (alias: CDX-1127), a fully humanised agonistic CD27 IgG1 mAb, in a phase 1 dose-escalation and -expansion trial, respectively was well tolerated (166). Only 1 patient experienced dose-limiting toxicity (grade 3 asymptomatic hyponatremia). Generally, pro-inflammatory immune activation was observed as characterised by increased terminally differentiated effector memory CD8^+^ T cells, HLA-DR expression on CD4+ T cells, and INFγ responses to recall antigens. One out of 15 RCC patients in the expansion cohort demonstrated a PR. Similarly, in hematologic cancers, varlilumab was tolerated well up to the maximum tested dose (167). 30 and 4 patients were tested in a dose-expansion and -escalation design, respectively of which 1 out of 4 Non-Hodgkin’s Lymphoma (NHL) patients developed CR. Both trials have shown modest clinical activity, pleading for the importance of combination therapies. Already assessed was the combination of varlilumab with nivolumab that could be applied safely in patients with several advanced solid tumours, not involving PLC (168). Though the combination regimen was not compared to nivolumab only, the ORR was not greater than expected for anti-PD-1 monotherapy. Results from a trial (NCT03396445) testing clinical efficacy of another fully humanised agonistic CD27 antibody (MK-5890) in a cross-over design comparing MK-5890 monotherapy to the combination with pembrolizumab (anti-PD-L1) are pending. To our knowledge, no data on treatment of PLC with CD27 targeting Abs are available yet.

#### 3.2.5 Type L receptor subfamily: CD40

CD40 (aliases: TNFRSF5, Bp50) is a co-stimulatory receptor that was discovered in 1986 (169). CD40 is constitutively expressed on APCs (e.g., macrophages, DCs, B cells) (170, 171). Furthermore, CD40 expression can be enhanced on fibroblasts as well as epithelial cells upon exposure to interferons (IFN) and tumour necrosis factor (TNF) (171, 172). In contrast to all other TFNRSF members, the orientation of CD40/CD40L on the DC:T cell synapse is inverted, with the receptor and ligand being expressed on the APC and T cell respectively, indicating a role in T cell priming rather than effector functionalities. CD40 ligand (CD40L; aliases: TNFSF5, CD154) serves as the sole ligand of the CD40 receptor (173). CD40L is primarily expressed by activated T cells and is upregulated upon TCR signalling (174). Under pro-inflammatory conditions, other immune cells that express CD40L are activated B cells, NK cells, mast cells, and basophils (175–178). CD40L expressing CD4^+^ T cells mainly interact with B cells in germinal centres and are therefore defined as Tfh cells (179). However, in immune oncology, CD40-mediated ICS is largely focused on the “licensing” of DCs allowing them to promote anti-tumour T cell activation and the skewing of macrophages (180). Generally, upon receptor crosslinking by CD40L, APCs upregulate major histocompatibility complex (MHC) molecules and co-stimulatory IgSF or TNFRSF ligands (e.g., CD70) (181). Moreover, CD40-activation induces secretion of cytokines that are crucial for CD8^+^ T cell activation as well as Th1 polarisation (e.g., IL12). By accomplishing enhanced antigen presentation and pro-inflammatory T cell support, CD40 activation potentiates anti-tumour immunity (181).

In HCC, CD40 is expressed on tumour infiltrating B cells and DCs. Total CD40 tumour expression correlated positively to improved survival, highlighting a significant role in anti-tumour immunity (159). Also in CCA, intra-tumoural CD40 expression was demonstrated to be an independent predictor for improved survival (182). However, *in situ*, the priming of anti-cancer immunity by APCs may be hampered in HCC, since intra-tumoural activated DCs showed less expression of CD40 when compared to adjacent tissues in the majority of patients, especially in those with tumour suppressor gene mutations (183, 184). Therefore, impaired DC maturation in HCC and CCA may prove to be a critical feature of tumour escape that offers therapeutic opportunity. Encouragingly, HCC PBMC-derived B cells transfected with HCC total RNA were able to induce cytotoxic T cell responses *ex vivo* upon activation with CD40L (185). Furthermore, CD40L-actived B cells of HCC patients were able to induce *in vitro* CD4^+^ and CD8^+^ responses in autologous TIL fractions to tumour-associated antigens (glypican-3, MAGE-C2) (18). These *in vitro* studies indicate that in HCC anti-tumour T cells can be induced when tumour antigens are effectively presented by well matured APC. As such there is potential for CD40 agonistic drugs in PLC. In four different CCA mouse models, treatment with an agonistic CD40 antibody alone achieved moderate anti-tumour immunity only (182). However, when combined with anti-PD-1 ICI, anti-tumour effects enhanced significantly by the induction of CD8^+^ T cell responses via activation of DCs and macrophages. Interestingly, anti-tumour activities were abrogated upon macrophage depletion, thus pointing out a critical role of myeloid cells for both CD40-mediated immunotherapy as well as effective anti-PD-1 therapy in CCA. These data highlight the possibility of targeting CD40 to facilitate T cell priming of *non-inflammed* tumours and support the combination of agonistic CD40-antibodies with T-cell targeting immunotherapy.

In 2007, selicrelumab (aliases: CP-870,893; RO7009789), a fully humanised agonistic CD40 IgG2 mAb, elicited a partial response in 4 out of 29 patients with advanced solid tumours. PR was observed in metastatic melanoma patients, and 1 out of 2 CCA patients experienced regression of a large hepatic metastasis (186). Even though, 55% of all study subjects developed grade 1-2 cytokine release syndrome (CRS) the safety profile was deemed acceptable. Similarly, in hematological cancer (B cell NHL), CD40 engagement via dacetuzumab (humanised agonistic CD40 IgG1 mAb; alias: SGN-40) was tolerated well amongst patients. Objective anti-tumour responses were reported in only a subset of patients (6 out of 50; 1 CR, 5 PR) (187). Other phase I and II trials on dacetuzumab in patients with multiple myeloma (MM) and diffuse large B-cell lymphoma (DLBCL), or on ChiLob7/4 (chimeric agonistic CD40 IgG1 mAb) in DLBCL patients have shown a similar safety profile as well as modest clinical activity (188–190). Recently, mitazalimumab, a fully humanised agonistic CD40 IgG1 (alias: ADC1013) has been shown to encompass a manageable safety profile among 95 patients with advanced solid tumours, unfortunately not including any PLC patients. Only 1 out of 95 patients (RCC) experienced partial response (191). These data have pleaded for identifying patients that are sensitive to CD40 engagement and again argue for combining CD40 agonistic antibodies with other regimen to enhance clinical activity. To enhance polarisation of macrophages to the pro-inflammatory M1 phenotype, sotigalimab (humised IgG1 agonistic CD40 antibody; alias: APX005M) was combined with inhibition of CSFR1 via cabiralizumab and co-administered with nivolumab (anti-PD-1) in patients with anti-PD-1/PD-L1-resistant melanoma, RCC, and NSCLC (192). Though the triplet therapy was tolerated reasonably and patient’s pharmacodynamics analysis suggested enhanced pro-inflammatory state, PR was reached in 1 out of 26 patients only. Lastly, in the phase 2 PRINCE trial studying nivolumab/chemotherapy, sotigalimab/chemotherapy, and nivolumab/sotigalimab/chemotherapy in metastatic PDAC patients, modest increase in OS has been observed in the nivolumab and sotigagalimab arms versus historical controls (193). However, only the nivolumab/chemotherapy arm met the primary study endpoint. Interestingly, analysis on PR-patients in the sotigalimab arm revealed that mainly genetic signatures related to CD4^+^ T cells, B cells, and DC subsets tend to predict for improved OS, in line with the envisioned mechanism of CD40 stimulation. Therefore, since CD40-mediated ICS induces T cell priming, agonistic CD40 antibodies should rather be used in combination regimen that offer tumour specific antigens as T cell targets or alleviate T cell suppression, like vaccination approaches or ICI respectively. Recent studies also suggests that we may not be looking at the right compartment to observe an immunomodulatory effect of the treatment. We may need to focus on tumour-draining lymph nodes for markers that predict response to therapy (194).

## 4 Enhancing ICS-mediated anti-tumour activity in PLC

### 4.1 Enhancing ICS-mediated anti-tumour activity in PLC requires a different approach compared to ICI

To date, the majority of phase 1 and 2 clinical trials on agonistic mAbs targeting immune co-stimulatory receptors as monotherapy or in combination with ICI have consistently shown no to modest anti-tumour activity only (**Table 2**). Though current regimens are tolerated well, none so far demonstrate enhanced clinical efficacy. Strategies to enhance ICS-mediated functionality either at low dosages or at higher dosages in a more tumour-restricted manner could aid to unlock the full therapeutic potential of ICS-mediated immunotherapy to enhance pre-existing *in situ* anti-tumour immunity. For PLC, the diverse repertoire of liver-resident immune cells and the expression of relating Fcγ receptors (FcγRs) on these cells provides unique opportunities to deliver enhanced on-target ICS.

**Table 2:** Immune co-stimulatory receptors are targeted using various antibodies in phase 1/2 clinical trials among various types of cancer.

Target receptor	Molecule	Ab isotype	Reference	Phase	Regimen	N	Tumour		DCR (%)			DLT (%)	Hepatoxicity (%)		
							Type	HCC/CCA	CR	PR	SD		Grade 1-2	Grade 3-4	
CD27	Varlilumab	hIgG1	(166)	1	Monotherapy	56	Metastatic solid tumours	-/-	0/56 (0%)	1/56 (2%)	8/56 (14%)	1/56 (2%)	-	
			(167)	1	Monotherapy	30	Refractory hematological tumours	-/-	1/34 (3%)	0/34 (0%)	4/34 (12%)	0/34 (0%)	1/34 (3%)	1/34 (3%)
			(168)	1	Nivolumab combination	36	Unresectable/metastatic solid tumours	-/-	0/36 (0%)	2/36 (6%)	11/36 (31%)	2/36 (6%)	4/36 (11%)	
				2	Nivolumab combination	139			1/139 (1%)	12/139 (9%)	37/139 (27%)	-	8/139 (6%)	
CD40	Selicrelumab	hIgG2	(186)	1	Monotherapy	29	Stage III/IV solid tumours	-/2	0/29 (0%)	4/29 (14%)	7/29 (24%)	3/29 (10%)	5/29 (17%)	2/29 (7%)
			(195)	1	Carboplatin/Paclitaxel combination	32	Advanced solid tumours	-/1	0/32 (0%)	6/32 (19%)	12/32 (40%)	2/32 (6%)	0/32 (0%)	1/32 (3%)
			(196)	1	Gemcitabine combination	21	Irresectible PDAC	NA	0/21 (0%)	4/21 (19%)	11/21 (52%)	-	-	
	Dacetuzumab	hIgG1	(187)	1	Monotherapy	50	Refractory/recurrent B cell NHL	NA	1/50 (2%)	5/50 (10%)	13/50 (26%)	2/50 (4%)	24/50 (48%)	2/50 (4%)
			(188)	1	Monotherapy	44	Refractory/recurrent multiple myeloma	NA	0/44 (0%)	0/44 (0%)	9/44 (20%)	6/44 (14%)	14/44 (32%)	4/44 (9%)
			(189)	2	Monotherapy	46	Refractory/recurrent DLBCL	NA	2/46 (4%)	2/46 (4%)	13/46 (28%)	-	18/46 (39%)	1/46 (2%)
			(190)	1	Monotherapy	29	Refractory solid tumours/DLBCL	-/-	0/29 (0%)	0/29 (0%)	15/29 (52%)	-	-	3/29 (10%)
	Mitazalimumab	hIgG1	(191)	1	Monotherapy	95	Advanced solid tumours	-/-	0/95 (0%)	1/95 (1%)	35/95 (37%)	2/95 (2%)	15/95 (16%)	5/95 (5%)
	Sotigalimab	hIgG1	(192)	1	Cabiralizumab/nivolumab comination	26	Anti-PD-1 resistant melanoma, RCC, NSCLC	NA	0/26 (0%)	1/26 (4%)	8/26 (31%)	1/26 (4%)	14/26 (54%)	11/26 (42%)
			(193)	1/2	Gemcitabine/nab-paclitaxel combination	36	Metastatic PDAC	NA	0/36 (0%)	12/36 (33%)	16/36 (44%)	-	12/36 (33%)	18/36 (50%)
					Gemcitabine/nab-paclitaxel/nivolumab combination	35			0/35 (0%)	11/35 (31%)	13/35 (37%)	-	11/35 (32%)	15/35 (43%)
OX40	9B12	mIgG1	(129)	1	Monotherapy	30	Refractory metastatic solid tumours	NR	0/35 (0%)	0/35 (0%)	25/30 (83%)	-	3/30 (10%)	0/30 (0%)
	Tavolimab	hIgG1	(130)	1	Monotherapy	55	Recurrent/metastatic solid tumours	1/-	0/50 (0%)	2/50 (4%)	22/50 (44%)	1/49 (2%)	0/55 (0%)	0/55 (0%)
			(29)	1	Neoadjuvant to surgical resection	17	Resectable HNSCC	NA	-			0/17 (0%)	-	
			(136)	1	Durvalumab combination	27	Refractory advanced solid tumours	0/1	0/21 (0%)	3/21 (14%)	9/21 (43%)	2/27 (7%)	5/27 (19%)	
					Tremelimumab combination	31		1/2	0/25 (0%)	0/25 (0%)	9/25 (36%)	3/31 (10%)	3/31 (10%)	
	BMS-986178	hIgG1	(28)	1/2	Monotherapy	20	Refractory/recurrent solid tumours	NR	0/20 (0%)	0/20 (0%)	7/20 (35%)	0/20 (0%)	-	
					Nivolumab combination	81			1/79 (1%)	5/79 (6%)	27/79 (34%)	0/43 (0%)	-	
					Ipilimumab combination	41			0/40 (0%)	0/40 (0%)	9/40 (23%)	0/35 (0%)	-	
					Nivolumab/Ipilimumab combination	23			0/23 (0%)	3/23 (13%)	12/23 (52%)	-	-	
	Ivuxolimab	hIgG2	(132)	1	Monotherapy	52	Advanced solid tumours	19/-	0/52 (0%)	3/52 (6%)	26/52 (50%)	0/52 (0%)	11 (21%)	
			(134)	1	Monotherapy	4	Refractory/recurrent AML	NA	-			0/4 (0%)	-	0/4 (0%)
			(137)	1	Utolimumab combination	57	Refractory advanced NSCLC, HNSCC, melanoma, UCC, Cervical cancer, GC	NA	0/57 (0%)	2/57 (4%)	18/57 (32%)	0/57 (0%)	-	
						30			0/30 (0%)	1/30 (3%)	14 (47%)	0/30 (0%)	-	
	ICAGN01949	hIgG1	(133)	1/2	Monotherapy	23	Refractory advanced solid tumours	2/2	0/87 (0%)	1/87 (1%)	23/87 (26%)	1/87 (1%)	-	
						64							-	
	GSK3174998	hIgG1	(135)	1	Monotherapy	45	Advanced/recurrent bladder cancer, CRC-MSI-H, HNSCC, melanoma, NSCLC, RCC, STS, TNBC	NA	0/45 (0%)	0/45 (0%)	4/45 (9%)	0/45 (0%)	-	
					Pembrolizumab combination	96			2/96 (2%)	4/96 (4%)	15/96 (16%)	2/96 (2%)	-	
			(138)	1	GSK1795091 combination	30	Refractory advanced solid tumours	NR	0/30 (0%)	1/30 (3%)	10/30 (33%)	1/30 (3%)	1/30 (3%)	
4-1BB	Urelumab	hIgG4	(26)	1/2	Monotherapy	346	Refractory advanced solid tumours	-/-	-	-	-	-	144/346 (42%)	44/346 (13%)
			(96)	1	Monotherapy	60	Refractory/recurrent hematological tumours	NA	3/60 (5%)	3/60 (5%)	11/60 (18%)	-	3/60 (5%)	1/60 (2%)
					Rituximab combination	46			4/46 (9%)	5/46 (11%)	10/46 (22%)	-	7/46 (15%)	1/46 (2%)
			(197)	1	Nivolumab combination adjuvant to SBRT	23	Refractory advanced solid tumours	-/1	-	-	-	0/23 (0%)	-	
	Utomilumab	hIgG2	(97)	1	Monotherapy	55	Advanced solid tumours/Merkel cell lymphoma	3/-	1/53 (2%)	1/53 (2%)	13/53 (25%)	0/55 (0%)	23/55 (42%)	0/55 (0%)
			(98)	1	Pembrolizumab combination	23	Refractory advanced solid tumours	-/-	2/23 (9%)	4/23 (17%)	10/23 (44%)	0/23 (0%)	-	
			(99)	1	Mogalizumab combination	24	PD-1/PD-L1 refractory advanced solid tumours	-/-	0/20 (0%)	1/20 (5%)	9/20 (45%)	0/24 (0%)	-	
			(198)	1	Rituximab combination	66	Refractory/recurrent hematological tumours	NA	4/66 (6%)	10/66 (15%)	28/66 (42%)	0/66 (0%)	16/66 (24%)	1/66 (2%)
			(100)	1	Avelumab/rituximab combination	9	Refractory/recurrent DLBCL	NA	0/9 (0%)	1/9 (11%)	1/9 (11%)	1/7 (14%)	-		
					Avelumab/azacitidine combination	9			0/9 (0%)	0/9 (0%)	0/9 (0%)	0/9 (0%)			
ICOS	Vopratelimab	hIgG1	(62)	1/2	Monotherapy	70	Refractory advanced solid tumours	-/2	1/70 (1%)		9/70 (13%)	2/70 (3%)	2/70 (3%)	2/70 (3%)
					Nivolumab combination	131		-/-	3/131 (2%)		27/131 (21%)	0/131 (0%)	6/131 (5%)	4/131 (3%)
	GSK3359609	hIgG4	(138)	1	GSK1795091 combination	11	Refractory advanced solid tumours	NR	0/11 (0%)	0/11 (0%)	2/11 (18%)	0/11 (0%)	-	
	MEDI-570	hIgG1	(199)	1	Monotherapy	23	Refractory T-NHL	NA	2/21 (10%)	5/21 (24%)	7/21 (33%)	0/23 (0%)	3/23 (13%)	1/23 (4%)
GITR	TRX-518	hIgG1	(30)	1	Monotherapy	43	Refractory solid tumours	2/1	0/43 (0%)	0/43 (0%)	4/43 (9%)	0/43 (0%)	-	
	MK-1248	hIgG4	(154)	1	Monotherapy	20	Refractory metastatic solid tumours	1/0	0/20 (0%)	0/20 (0%)	3/20 (15%)	0/20 (0%)	-	
					Pembrolizumab combination	17		-/-	1/17 (6%)	2/17 (12%)	5/17 (29%)	0/17 (0%)		
	BMS-986156	hIgG1	(27)	1/2	Monotherapy	34	Refractory advanced solid tumours	-/-	0/34 (0%)	0/34 (0%)	11/34 (32%)	0/34 (0%)	0/34 (0%)	0/34 (0%)
					Nivolumab combination	258		14/-	2/258 (1%)	19/258 (7%)	84/258 (33%)	1/258 (1%)	3/258 (1%)	2/258 (1%)
	MK-4166	hIgG1	(155)	1	Monotherapy	48	Metastatic solid tumours	-/-	0%	0%	23%	1/48 (2%)	-	
					Pembrolizumab combination	65		1/-	0%	2%	25%	0/65 (0%)		
	GWN323	hIgG1	(156)	1	Monotherapy	39	Advanced solid tumours/lymphomas	-/-	0/39 (0%)	0/39 (0%)	7/39 (18%)	0/39 (0%)	-	0/39 (0%)
					Spartalizumab combination	53		-/-	1/53 (2%)	3/53 (6%)	14/53 (26%)	3/53 (6%)	-	6/53 (11%)

AML, acute myeloid leukemia; CCA, cholangiocarcinoma; CR, complete response; CRC, colorectal carcinoma; DCR, disease control rate; DLBCL, diffuse large B cell lymphoma; DLT, drug-related toxicity; GC, gastric cancer; HCC, hepatocellular carcinoma; hIg, humanized immunoglobulin; HNSCC, head and neck squamous cell carcinoma; mIg, mouse immunoglobulin; NA, not applicable; NHL, non-Hodgkin lymphoma; NR, not-reported; NSCLC, non-squamous cell lung carcinoma; PDAC, pancreatic ductal adenocarcinoma; PD1, programmed death-1; PR, partial response; RCC, renal cell carcinoma; SD, stable disease; STS, soft tissue sarcoma; TNBC, triple negative breast cancer; UCC, urothelial cell carcinoma.

#### 4.1.1 Translating principal differences of agonistic ICS Abs compared to antagonistic ICI Abs

Up to now, much clinical experience on anti-cancer immunotherapy has been obtained from large cohort studies. However, these studies have primarily focused on ICI using antagonising mAb. In contrast, high affinity towards cognate epitopes as well as ligand competition are of less importance to agonistic ICS approaches and this needs to be considered when developing such strategies.

#### 4.1.2 Receptor super clustering is essential for downstream signaling of ICS

By nature, CD28-, ICOS-, and TNFRSF-mediated ICS signaling requires clustering or higher-order oligomerisation of costimulatory receptors: receptor super clustering (200). For example, soluble TNFRSF ligands demonstrate reduced agonistic capacities compared to membrane bound forms. A key factor for optimal ICS following agonistic binding is the potential of the mAb for multivalent binding and induction of receptor super clustering (201). Natural IgSF ligands (e.g., B7H1, ICOSL) configure as homodimers that account for bivalent engagement of separate singular IgSF receptors. Moreover, natural TNF ligands mostly present as tertiary confirmations that engage with cognate receptors in receptor-trimer complexes (i.e., 3:3 configuration). Receptor complex multimerisation has been demonstrated to greatly enhance co-stimulatory receptor activation. Preclinical and clinical trials have proven the synergistic effect of receptor clustering through the application of receptor ligands as well as multivalent ligands compared to monomeric signaling (202–204). Multivalent agonistic aptamers directed to OX40 and 4-1BB have shown to induce greater TNFRSF activation (205, 206). *In vitro* activation of HCC-derived TILs was enhanced upon exposure to multimeric OX40L and GITRL, whereas monomeric Abs did not or hardly stimulate CD4^+^ and CD8^+^ T cell proliferation, respectively (126, 152). Concordantly, whenever OX40 mAbs were coupled to beads, creating multimeric anti-OX40 IgG2, TIL expansion increased. These results highlight the significant role of TNFRSF multimerisation for activating TILs in HCC. Consequently, current agonistic ICS mAb may establish suboptimal receptor super clustering and this should be optimised to enhance ICS-mediated T cell activation.

#### 4.1.3 Enhancement of FcγRIIB affinity increases the agonist potential of mAb through multimerisation

**Figure 3:**
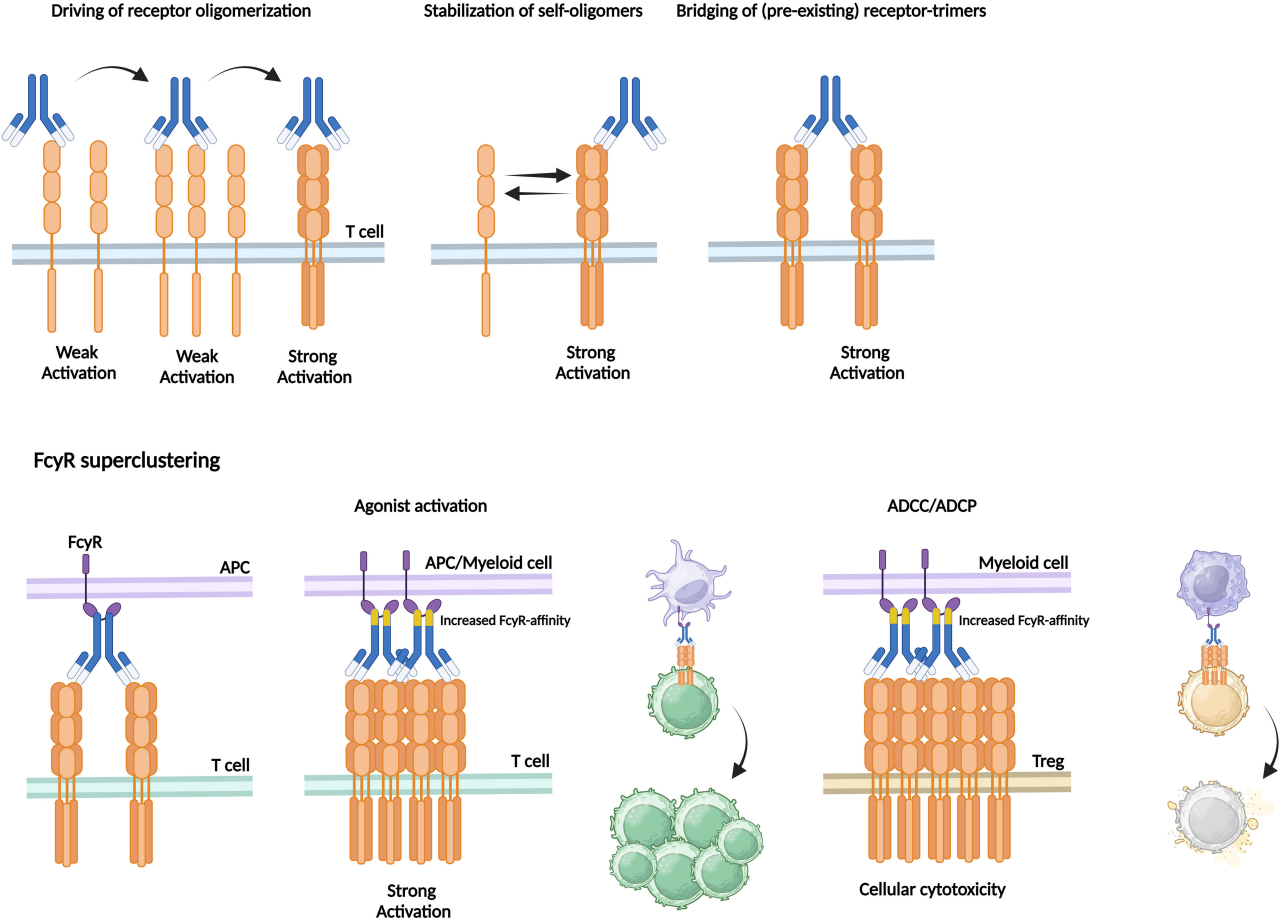
Agonistic mAb require receptor oligomerisation as can be enhanced by increased FcγR-affinity. To induce strong activation, agonistic mAb should either actively drive receptor oligomerisation, stabilise self-assembled receptor oligomers, or bridge between pre-existing receptor-trimers. Receptor-trimer bridging is achieved via FcγR-mediated receptor crosslinking. FcγR-expressing APCs bind to the Fc-region of the antibody that is bound to the target receptor expressed by T cells. Increased FcγR-affinity can enhance the extent of FcγR superclustering thereby inducing strong T cell activation followed by proliferation or enhanced ADCC/ADCP-mediated cellular cytotoxicity of for in stance immunosuppressive Tregs.

Natural IgG is capable of bridging multiple receptors via the two connected antigen-binding fragments (Fab) that induce monomer-monomer or oligomer interactions albeit insufficient to support proper ICS (207). Agonistic mAb should either actively drive receptor oligomerisation, stabilise self-assembled receptor oligomers, or bridge between pre-existing receptor-trimers (**Figure 3**). Receptor-trimer bridging can be achieved using FcγR-mediated receptor crosslinking. FcγRs bind the constant crystallizable fragment (Fc) domain of IgGs, and depending of the subtypes this binding induces activating or inhibitory signals regulating the function of various immune subsets (208). In human, six different FcγRs have been described of which 5 immune-activating (high affinity immunoglobulin-γ FcRI, FcγRI; low affinity immunoglobulin- γ FcRIIa, FcγRIIA; low affinity immunoglobulin-γ FcRIIc, FcγRIIC; low affinity immunoglobulin-γ FcRIIIa, FcγIIIA; low affinity immunoglobulin-γ FcRIIIb, FcγIIIB) and 1 inhibitory variant (FcγRIIB) (208). The inhibitory FcγRIIB is expressed on APC and myeloid subsets (DC, macrophage, B cell, NK cell, etc.) and uniquely functions as a scaffold crosslinking IgGs which can be exploited to crosslink also agonistic mAb (208).

In HCC-derived TILs FcγRIIB is widely expressed, facilitating the FcγRIIB-dependent effect of any agonistic ICS mAb. The majority of intra-tumoural B cells and cDCs express FcγRIIB and to a smaller extent it is expressed on monocytes and NK cells (126). Binding to inhibitory FcγRIIB has been widely demonstrated to be of utmost importance for agonistic mAbs to drive potent CD28-, CD40-, OX40-, and 4-1BB-mediated ICS via receptor trimerisation (209–211). After mAb engage with their cognate TNFRSF epitope their Fc domains are captured by FcγRIIB-expressing APCs or myeloid cells forming a scaffold facilitating TNFRSF clustering and activation *in trans*. Accordingly, Campos-Carrascosa and colleagues treated HCC-derived TILs with an Fc-engineered αOX40 human IgG1 antibody, termed αOX40_v12, that contained six mutations leading to increased affinity to FcγRIIB (E223D, G237D, H268D, P271G, Y296D, A330R). When compared to the native agonistic αOX40 human IgG1 antibody αOX40_v12 appeared to improve *in vitro* TIL expansion and functionality. These novel generation Fc-engineered agonistic mAbs are currently widely studied (126, 212, 213).

#### 4.1.4 Enhancement of activating FcγR engagement potentiates ADCC/ADCP-mediated anti-tumour activity of ICS agonistic Abs

In contrast to current ICI that relies mostly on direct T cell activation, agonistic ICS mAbs may also stimulate CD8^+^ effector T cells indirectly by the depletion of immunosuppressive cells (e.g., Treg). Antibodies binding to activating FcγRs expressed by NK cells, macrophages, or granulocytes, can trigger cell-mediated cytotoxic effector functions such as antibody-dependent cellular cytotoxicity (ADCC) and phagocytosis (ADCP) (214, 215). Upon mAb-antigen recognition, target cells are opsonised by antibodies, recognised by activating FcγR expressing cells and subsequently directly killed or phagocytosed by these cells. In human, FcγR3, expressed on NK cells and monocytes, is considered the primary activating receptor driving ADCC (216).

Whereas inhibitory FcγR2B engagement stimulates downstream signaling through receptor multimerisation, activating FcγR binding facilitates ADCC and ADCP activities of agonist ICS mAbs (157). Notably, engagement by FcγR2B reduces antibody availability for activating FcγRs (217). Therefore, IgG isotype selection is critical for the design of ICS mAb therapies. IgG1 has highest affinity to all activating FcγRs, whereas IgG2 and IgG4 only bind moderately to FcγRII and FcγR1 (IgG4 only) (216). mAbs with so called high activating: inhibitory (A:I) ratios tend to have greater cell-mediated cytotoxic effector functions, but lower agonistic activity (218). To establish effective anti-tumour immunity through ICS mAbs (esp. of the IgG1 isotype), the relative effect of immune cell activation *versus* immune cell depletion is likely determined by the tumour’s immune context.

As the TIME of PLC is highly enriched by immunosuppressive Tregs that hamper cytotoxic T cell-mediated anti-tumour immunity, greater ADCC effects might be desired rather than direct immune cell activation. Both HCC- and CCA-derived tumour infiltrating Tregs express high levels of co-stimulatory IgSF (ICOS) and TNFRSF (4-1BB, OX40, GITR) members (18, 55, 91, 92, 124, 126). As such, agonistic ICS mAbs of the IgG1 isotype might be of particular interest in depleting immune suppressive Tregs through ADCC mediated by liver resident Kupffer cells and NK cells that express relative high levels of FcγRIII (219, 220). Similar to discussed Fc-engineering strategies to increase binding to FcγRIIB, approaches to specifically enhance binding of the Fc domain to activating FcγRs could be employed to potentiate ADCC/ADCP-mediated anti-tumour activity of ICS agonistic Abs. Full anti-tumour immunity is however likely not to be expected from depletion of immunosuppressive subsets alone. Among various solid tumours in human, including HCC and CCA, Treg depletion by GITR agonism did increase Teff: Treg ratios, but was not sufficient to activate cytolytic cells due to persistent *in situ* TIL exhaustion (30). Accordingly, cytotoxic T cell re-invigoration in PLC might be supported by depletion of Tregs or any other immunosuppressing subsets but should likely be supported by ICS-mediated T cell activation and ICI in the appropriate dosing-regimen as well.

#### 4.1.5 Agonist ICS mAbs require different dosing-regimen compared to ICI mAbs

In contrast to conventional ICI mAbs, ICS demonstrate a variable dose-response relationship. Classical mAbs primarily achieve clinical activity through receptor antagonism or ADCC, ADCP, and complement-dependent cytotoxicity (CDC). These modes of action establish optimal functionality at binding saturation of the cognate receptors. At peak receptor occupancy, increased dosage concentration will not induce any additional effect thereby reaching a plateau (201). In contrast, preclinical *in vitro* studies on ICS have shown clinical activity to decrease after a peak at specific concentrations has been reached (221, 222). Thus, agonistic ICS mAbs act in a *bell-shaped* dose-response rather than the classical *sigmoidal* dose-response functionality.

This mechanism might be partly attributed to the mAbs’ stoichiometric binding properties to the cognate receptors (201, 222). Theoretically, formation of maximal receptor superclustering should be achieved upon bisected molar concentrations of antibody to receptor thereby providing optimal bridging between the both. If antibody or receptor abundance exceed each other, inadequate antibody-receptor bridging would be established leading to isolated complexes with a 2:1 or 1:2 stoichiometry, respectively. At the dose-optimum, effective ICS-mediated T cell activation can be solely established directly via maximal receptor multimerisation. Therefore, optimal dosing strategies may require adaptations based on biomarkers (e.g., T cell proliferation or activation) to carefully monitor these dynamics.

Secondly, optimal activity at a certain dose-optimum might be explained by dynamical T cell functionality as well. Prolonged T cell activation through chronic antigen exposure via ICS-supported TCR signaling drives immune cell exhaustion leading to downregulation and activation-induced cell death. Whereas ICI mAbs disinhibit T cells that have been primed already, high dose agonistic ICS mAb concentrations could facilitate substantial T cell priming and overstimulation favouring subsequent T cell exhaustion. Combination regimen incorporating ICI might be a logical consideration to revert T cell exhaustion. However, albeit administered concomitantly rather than sequentially, various clinical trials failed to show any additional effect of ICS/ICI combination therapy over ICS monotherapies (27, 28, 135, 136, 154, 168). Therefore, better understanding of the process of T cell differentiation; in particular on the relation between priming, activation and exhaustion, is crucial. Effective combination regimen should preferentially first apply ICS agonists to enable tumour-specific T cell activation possibly even towards exhaustion. As activation and exhaustion are characterised by enhanced co-inhibitory receptor expression, this approach should then be followed by ICI mAbs to prevent suppression via co-inhibitory receptor ligands in the TIME (215, 223).

Other than ICI that bind to broadly expressed co-inhibitory receptors, co-stimulatory receptor expression might be relatively low on effector TILs, particularly on the cytotoxic immune compartments. As mentioned previously, co-stimulatory receptors are expressed fairly transiently on activated CD4^+^ and CD8^+^ T cells. However, HCC- and CCA-derived TILs demonstrate relatively lower expression of ICOS, OX40, and GITR on CD8^+^ T and (a)Th cells when compared to (a)Tregs (18, 126, 152). and thus it might be required to re-prime TILs prior to administration of any agonistic ICS Ab (combination) regimen. Strikingly, the pre-activation status of TILs correlated positively to *ex vivo* response rates upon OX40-mediated ICS (126). Several strategies could be of use here. i.e., toll-like receptor (TLR) agonists, vaccination, radiation or low dose metronomic chemotherapy to achieve immunogenic cell death. In HCC, previous locoregional therapies demonstrate to enhance Ki67 expression in HCC-derived TILs (126, 224). Thus, these data support the concept of *priming* the TIME prior to ICS-mediated anti-tumour immunity in PLC.

### 4.2 Tumour-targeted delivery of ICS through bispecific antibody approaches can reduce off-target (hepatotoxic) effects

Although, agonistic ICS mAbs aim to enhance anti-tumour functionality, they have been demonstrated to cause treatment-related immune-mediated adverse events. To some extent, prior priming of immune cells using locoregional therapies might direct immune activation towards the TIME. However, these effects may extend to non-tumourous surrounding tissues as well, potentially contributing to severe organ damage. Therefore, more tumour-restricted delivery of immune activation may be warranted by obligate bispecific antibodies (bsAbs).

Engineered IgG-like bsAbs have a single IgG incorporating two Fab arms that have distinct antigen specificities and can therefore be directed to distinct receptors (225). Physical linkage of two binding specificities warrants a dependency that can be either spatial (in-trans) or temporal (in-cis) (226). In-trans binding redirects effector T cell cytotoxicity to specifically eliminate target cells by linking T cells with tumour cells to form an immune synapse via a T-cell and tumour-binding domain. Similarly, ICS agonists functionality can be directed to the TIME using tumour-restricted antigen thereby increasing therapeutic efficacy and minimising any off-target ICS activities. Moreover, application of an ICS binding domain will not only attract T cells to the tumour site, but co-stimulatory receptor expressing NK cells as well, potentially leading to NK cell mediated toxicity. In-cis binding bi-specific antibodies co-targeting 2 receptors on the same cells may be used to restrict ICS activation to tumour-reactive T cells. Binding to distinct receptors would allow to simultaneously block two pathways (antagonist-antagonist pairing; e.g., PD-1 and CTLA-4) or pair antagonist to agonist (e.g., PD-1 to 4-1BB) and agonist to agonist (e.g., OX40 to 4-1BB). Though dual antagonist pairing (i.e., PD-1 x LAG3 and PD-1 x TIM3) seems to robustly enhance anti-tumour activity in the preclinical setting, heterodimerisation of different co-stimulatory receptors has to be studied in more detail (227–232). Intriguingly, some TNFRSF members have been demonstrated to signal as mixed oligomers (233). As TNFRSF generally engage shared downstream TRAF adaptor proteins, simultaneous receptor binding of bsAbs to distinct co-stimulatory receptors might allow for downstream ICS signaling that is equally effective as homodimerisation of individual receptors, albeit in a more tumour-specific manner.

In PLC, bsAbs have great potential in tumour-restricted delivery of ICS-mediated activation. Incorporation of a binding epitope directed to either tumour-associated antigen (TAA) or tumour-specific neoantigen (neoAg) might direct tumour cell targeting. Tumour-associated antigens (TAA) feature non-mutated amino acid sequences that are enriched within cancer cells, but may be presented by HLA on the surface of non-malignant cells as well. In HCC, TAA compromise oncofetal and cancer-germline antigens such as glypican 3 (GPC3) and melanoma-associated gene C1 (MAGE-C1) (234, 235). In CCA patients, TAA have been described in small cohorts only (236) and evidence on TAA-mediated oncogenicity and systemic TAA-reactive immune responses remains limited (128). However, in metastasised CCA patients Löffler and colleagues reported efficient tumour immune cell infiltration upon TAA-peptide vaccination in CCA metastatic lesions, suggesting the pre-existence of a TAA-reactive immune cell repertoire in these patients (237). bsAbs that target oncofetal protein GPC3 and 4-1BB are already in preclinical development (PRS-342) and might be promising candidates in directing and stimulating recently activated 4-1BB^+^ TILs to HCC tumour tissues. Lastly, dual receptor engagement might restrict ICS to tumour reactive HCC-derived TILs. As these subsets were described to be delineated by the expression of 4-1BB and PD1, bsAbs targeting both receptors could potentially specifically enhance anti-tumour immune activities (92).

## 5 Future application of ICS-mediated agonistic Abs in PLC management and conclusions

Though current immunotherapies for PLC have clearly shown to have some clinical effects, ICI-mediated antagonist antibodies reach clinical anti-tumour efficacy in a minor subset of advanced-stage HCC and CCA patients only (12, 15). Multi-facetted approaches addressing cytotoxic as well as immunosuppressive elements of the PLC TIME seem to improve anti-tumour immune activation (238). Therefore, attention and expectations have shifted towards combination treatments incorporating anti-PD-1, -PD-L1, and -CTLA-4 mAbs rather than single-agent ICI-regimen. In PLC, co-stimulatory receptors are widely expressed among immune regulatory and activatory cell subsets, thereby potentially facilitating reinvigoration of *in situ* anti-tumour activity in a dual manner.

When applied properly, ICS-mediated immune activation might hold great promise in enhancing the pool of HCC and CCA ‘responders’ at various stages of disease. As the HCC- and CCA-derived TIME are highly enriched for immunosuppressive cell subsets, single-agent ICS-regimen should primarily aim for the enhancement of A:I ratios and subsequent ADCC and ADCP functionalities. Elimination of Treg function appears crucial as the suppressive capacity of Tregs is potentially enhanced upon current anti-PD-L1 ICI regimen (239). Intra-tumoural Treg removal might allow more efficient activation of cytotoxic CD8^+^ T cells, either via direct ICS alone or in combination with *in situ* (ICS-supported) vaccination followed by previous ICI approaches.

Specifically in PLC, the use of new generation ICS-mediated Abs should be investigated to enhance binding to activating FcyRs to promote ADCC/ADCP. Given FcyRIIB-mediated inhibition of ADCC, minimal to no engagement to inhibitory FcyRs should be strived at. Alternative approaches to enhance co-stimulatory receptor multimerisation could still consist of biAbs or state-of-the-art Fc-coupled fusion proteins (240). Moreover, adequate dosing regimen should be determined for patients taking *in situ* TIL activation status into account. Application of such optimised ICS-mediated single- or combination-regimen might potentially make the HCC and CCA-derived TIME more susceptible to immunotherapies in advanced-stage disease.

## Author contributions

YR: Conceptualization, Data curation, Formal analysis, Investigation, Methodology, Project administration, Resources, Validation, Visualization, Writing – original draft, Writing – review & editing. SB: Conceptualization, Funding acquisition, Methodology, Resources, Supervision, Writing – original draft, Writing – review & editing. JI: Conceptualization, Funding acquisition, Methodology, Resources, Supervision, Writing – original draft, Writing – review & editing. DS: Conceptualization, Funding acquisition, Methodology, Resources, Supervision, Writing – original draft, Writing – review & editing.
